# Primary Lateral Sclerosis

**Published:** 1897-02

**Authors:** S. G. Courtney Pinckney

**Affiliations:** Atlanta, Ga.; Lecturer on Diseases of the Mind and Nervous System, Southern Medical College; 302 Peachtree Street


### PRIMARY LATERAL SCLEROSIS.

BY S. G. COURTNEY PINCKNEY, M. D., ATLANTA, GA.

Lecturer on Diseases of the Mind and Nervous System, Southern Medical College.

Although autopsy has failed in some cases to show the pathological condition suggested by Erb and Charcot as the cause of this variety of spastic paraplegia, still a degeneration of the pyramidal tracts has been pretty constantly found; doubt exists only as to whether a primary degeneration limited to the motor tracts occurs as separate system disease.

In nearly all the cases so far examined, the pyramidal degeneration has been combined with other lesions and has been apparently secondary to them—transverse myelitis, hydromyelus, cerebellar tumor, chronic basilar meningitis, etc. Still more frequently the anterior grey horns have shown cell degeneration, and although this may not have advanced far enough to cause atrophic muscle changes, we must consider these cases as an undeveloped variety of amyotrophic lateral sclerosis.

In all these cases mentioned the symptoms were those of a primary uncomplicated degeneration of the pyramidal tracts' hence the doubt as to whether some such condition is not present in cases that have not come to autopsy.

In at least two cases the condition diagnosed was present without other changes, therefore we must admit its existence as a special disease.

Clinically the symptoms are clear enough. The slowly increasing spastic paraplegia without sensory, bladder or rectal symptoms is not apt to be mistaken. A lumbar myelitis is the nearest approach to it in appearance, and the fact that such a myelitis may at first be entirely confined to the motor columns prevents a positive diagnosis until the disease has existed at least a year; then if neither sensory nor bladder symptoms have appeared we can, with fair certainty, exclude that.

The etiology too, gives us but little aid, it being that common to most cord disease. Sudden cooling of the skin was the probable cause in three of the eight cases of which I have notes two of the patients being bakers—the other a stoker on a steamship. In these cases the onset was rather subacute, coming on after going into the cold air from a superheated room. The disease was fairly well developed in a few weeks—a more sudden onset than I have ever seen described.

Of the other cases the probable causes were: A severe fall on the back in one case; continued exposure to wet cold in one other, and muscular strain in a third. In two cases no apparent cause could be found.

It is noticeable that only in one case could a history of syphilis be obtained, and then it seemed to play no part in the disease. All these cases were males—the youngest 22—the eldest 56 years. The greatest liability seemed to be between 25 and 35, all the other cases being in that decade.

In several other cases in which a provisional diagnosis of primary lateral sclerosis was made the consequent appearance of bulbar, sensory, bladder or trophic symptoms removed them to another category. In these cases mentioned, no cause for change was found at the end of two years.

Treatment seems to be markedly unsatisfactory—the cases showed a steady increase of symptoms. Great temporary improvement followed systematic rest; two weeks in bed resulted in decidedly lessening the rigidity for several months; immersion in a very warm bath for an hour gives temporary relief. Drugs, electricity and suspension were useless.

*302 Peachtree Street.*

